# Exploring EFs and Math Abilities in Highly Deprived Contexts

**DOI:** 10.3389/fpsyg.2020.00383

**Published:** 2020-03-10

**Authors:** Sandra Pellizzoni, Gian Matteo Apuzzo, Chiara De Vita, Tiziano Agostini, Miriam Ambrosini, Maria Chiara Passolunghi

**Affiliations:** ^1^Department of Life Sciences, University of Trieste, Trieste, Italy; ^2^Terre des Hommes, Milan, Italy

**Keywords:** executive functions, working memory, inhibitory control, war context, early math abilities, preschoolers

## Abstract

Executive functions (EFs)’ development is critically affected by childhood adversity exposure. Although recent studies underlined the deleterious effects of early life stresses on working memory (WM) and inhibitory control, they were scarcely investigated in war context especially in relation with learning abilities. In order to fill this gap, we designed a research with the aim to evaluate EFs together with early math skills. In particular, we conducted a study involving 150 children divided into three groups: 48 Yazidis (*M*_age_ = 71 months, *SD* = 6.59), 47 Syrian refugees (*M*_age_ = 68.77 months, *SD* = 7, age), and 55 Italians (*M*_age_ = 68.65 months, *SD* = 2.88) attending the third year of kindergarten in Italy or inserted in Psyco-Social-Support activities in Iraq. The children were evaluated with a variety of tasks assessing WM, inhibitory control, counting, digit-quantity mapping, and digit naming skills. The results indicated substantial differences both in EFs and early numerical abilities between the deprived groups and the Italian children. Data are discussed in terms of implications for children both exposed to mainstream school environments and living in socio-economically disadvantaged and deprived contexts.

## Introduction

Early exposure to deprived environments, deviating considerably from the care that is typical for children, may represent a risk factor for negative longer term outcomes and lasting alterations at both cognitive, social–emotional, and behavioral level (Merz et al., 2016). Experience-expectant models of development suggest that, for typical neural development to proceed, expected environmental input, such as the presence of a sensitive and responsive attachment figure, adequate physical resources (e.g., nutrition) as well as social and linguistic stimulation matched to child’s developmental stages and needs, must be provided at certain sensitive periods (Marshall and Kenney, 2009). A recent review of the literature confirms that early adverse experiences get “under the skin” with specific effects on hypothalamic–pituitary–adrenal axis, level of inflammation and brain functioning (Danese and McEwen, 2012). More specifically, in relation to the brain activities, literature shows the effect of maltreatment and early stress during childhood (McCrory et al., 2011) on three areas known to be highly sensitive to psychosocial stress: amygdala, hippocampus, and prefrontal cortex (PFC). PCF, in particular, is considered the neural substrate of executive functions (EFs) (McEwen and Morrison, 2013).

Executive functions are defined as a set of interrelated top-down mental processes crucial for goal-directed activities. These functions allow individual to (1) hold, update, and actively manipulate information in mind, (2) inhibit inappropriate responses, (3) show flexibility in strategies, ideas, and activities (see Zelazo and Müller, 2002; Miyake and Friedman, 2012). EFs have been extensively investigated in children (for a review see Garon et al., 2008), resulting significantly related to learning abilities on both reading and math, overall school achievement (Blair and Razza, 2007; Bull et al., 2008; Clark et al., 2010; Bull and Lee, 2014), as well as to later academic outcomes (e.g., McClelland et al., 2014). By means of a cascade effect, EFs are broadly considered fundamental for the acquisition and mastering of more complex skills, such as early mathematical abilities (e.g., Espy et al., 2004; Bull et al., 2008). More specifically, previous studies conducted on typically developing children showed that WM skills predicted numerical competence in preschool and primary school both directly (e.g., Bull et al., 2008; Passolunghi and Lanfranchi, 2012) and indirectly (e.g., Krajewski and Schneider, 2009). Similarly, inhibitory control during the preschool years accounted for variability in children’s early math achievement 1 year after school entry (see Clark et al., 2010).

Taken together, these pieces of information seem to indicate that early adversity crucially undermines domain-general skills (i.e., EFs) which, in turn, are associated with achievement abilities. Studies on the field confirm that institutionalized children show poorer levels of inhibitory control and WM ability (Merz et al., 2016), as well as children diagnosed with maltreatment-related post-traumatic stress disorder (PTSD) reveal more distractibility and lower sustained visual attention (Beers and De Bellis, 2002). Furthermore, familial trauma is shown to have an effect on EFs’ composite score, including performance in WM, inhibition, auditory attention, and processing speed tasks (DePrince et al., 2009).

However, literature on EFs is surprisingly lacking on children living in war-affected contexts, and related refugee conditions, despite the enormous relevance and topicality of these issues in contemporary society. In fact, approximately one in six children today lives in a war context (Save the Children International, 2018) as well as a huge number of children (seventy-one million, UNHCR, 2019) is currently displaced in the world and at a potential risk of cognitive disadvantage and mental illness. Considering this state of art, it is critical to organize evidence-based interventions targeted for specific deprived life conditions.

A recent study on Yazidi children indicated that preschoolers, living in a critically adverse context, show lower scores in hot and cool EFs tasks, in particular in delay of gratification and inhibition abilities, with a specific effect on motor (circle drawing task) and prevalent response (day and night Stroop task) control (Pellizzoni et al., 2019), thus confirming previous developmental research (Merz et al., 2016). On the other hand, a study conducted by Chen et al. (2019) underlines a specific effect of poverty, but not violence, on WM. These findings highlight the importance of carefully distinguishing between types of childhood adversity exposure (e.g., violence and poverty) in order to identify the specific relevant neurocognitive pathways underlying children’s cognitive functioning as well as their psychosocial well-being.

In the light of this state of art, the aim of this study was to explore the signature of living in war context investigating EFs and math abilities in three groups of preschool children Italian, Syrian and Yazidis. In particular we expected to: (a) confirm the relation between deprived living environments, specifically focusing on genocide context and refugee condition, and poor EFs, already found in literature (e.g., Welsh et al., 2010; Merz et al., 2016; Pellizzoni et al., 2019); (b) observe lower early mathematical skills in deprived children, comparing three groups of preschoolers coming from different socio-cultural and economic backgrounds (i.e., Yazidis, Syrian refugees, and Italians).

## Materials and Methods

### Participants

Participants were 150 children divided into three groups: 48 Yazidis (*M*_age_ = 71 months, *SD* = 6.59, age range: 62–80 months, 24 females), 47 Syrian refugees (*M*_age_ = 68.77 months, *SD* = 7, age range: 60–80 months, 24 females), and 55 Italians (*M*_age_ = 68.65 months, *SD* = 2.88, age range: 62–72 months, 28 females). Yazidis are a Kurdish religious minority found primarily in northern Iraq, southeastern Turkey, and northern Syria; the majority of Yazidis live in northern Iraq and they have suffered numerous atrocities perpetrated by ISIS that are described as genocide (United Nations Human Rights Council, 2016, June 15, 32nd session); most of them are currently internally displaced people (IDPs). On the other hand, the sample of refugees included children from different areas of Syria living in refugee camps specifically organized to accommodate them. The data were collected in the HARSHAM Camp, close to the city of Erbil, that hosts mainly Syrian refugees together with internal displaced persons (IDPs), and in the Bajid Kandala Camp, in the Dohuk Governorate closed to the border with Syria and Turkey, hosting mainly IDPs belonging to Yazidis’ community, who survived the genocide. Both Camp facilities include tents and prefabricated shelters and containers, and they offer internal activities destined to preschoolers focused on promoting aggregation and socialization among the children and including play together, respect simple roles, painting, and enhancement of motor abilities. Italian children were recruited from three preschools located in different urban areas of northern Italy, serving middle socioeconomic status families. None of the participants displayed developmental delay or reported learning difficulties.

### Procedure

Consent to participate in the research was obtained from children’ teachers and parents and participants also gave verbal assent before being tested. Children’s assessment was conducted in a single session, lasting approximately 30 min, in a quiet space. The order of tasks presentation was counterbalanced across participants. In the Italian sample, evaluation was carried out by two experimenters (female Italian master students), while in the other two cases (i.e., Yazidis’ and Syrian refugees’ groups) testing was guided by two social workers (one male and one female) in Arabic (for Syrian refugees) and Kurdish Badini (for Yazidis).

In math-related use of words, the Arabic and Italian languages have a very similar structure: for example, the term naming the number 11 (eleven) includes both components, ten + one; therefore, in these two cases, the use of the number words has a quite regular structure. Yazidi minority group use – both as spoken and written language – the Kurdish Badini, though they know and speak both Arabic and other variants of Kurdish. Given the fact that children were less familiar with Arabic than with Badini, and in order not to create other potentially confounding factors associated with less frequent use of Arabic language, Yazidi children were tested in Badini. The latter has completely novel words for 11 and 15 while the other number words have a regular structure.

The data collectors were from the same language/cultural background of the children, and native speakers of the participants’ language. In order to guarantee a reliability between data collectors, we provided a training on the consequences of trauma on behavior and cognitive abilities, specifically on EFs, and described tools that could provide a specific cognitive evaluation. Moreover, data collectors were trained directly in the field, supervised by an expert researcher, on how to evaluate a child and how to report the results.

### Measures

#### Executive Functions

##### Working memory

Working memory skills were measured using the backward word span task (adapted from Lanfranchi et al., 2004). Participants were read lists of two to five words and were required to recall each sequence in reverse order to that used by the examiner. The test included four difficulty levels, for a total of eight trials. A score of one was given for each sequence correctly recalled (expected range 0–8). The test–retest reliability was 0.84.

##### Inhibitory control

Inhibition skills were tested using the day and night Stroop task (Gerstadt et al., 1994), comprising a congruent and an incongruent (or Stroop) condition. In each condition children were shown a sequence of 16 pictures presented one at a time, eight depicting the sun and eight depicting the moon. In the congruent condition, children were asked to say either “day” or “night” whenever a picture of the sun or the moon was presented, respectively. In the incongruent condition, participants were required to say “day” for the picture of the moon and “night” for the picture of the sun. One point was given for each correct response in each condition (expected range 0–16). The test–retest reliability was 0.97 for the incongruent condition.

#### Early Mathematical Abilities

##### Forward counting

To measure forward-counting skills, we used a task adapted from the forward sequence subtest of the Numerical Intelligence Battery (BIN; Molin et al., 2007). Children were asked to recite aloud the numerical sequence from 1 to 50 and obtained one point for each correct response. Considering the differences between the number word systems of Italians and Syrians on the one hand, and Yazidis on the other, in order to examine distinctly performance for single digits and two-digit numbers, we analyzed forward counting separately for 1–10 and 11–50 (expected ranges 0–10 and 0–40, respectively). The test-retest reliability was 0.83 for counting from 1 to 10 and 0.82 for counting from 11 to 50.

##### Backward counting

Backward-counting skills were tested using a task adapted from the backward sequence subtest of the BIN (Molin et al., 2007). Participants had to recite the numerical sequence backwards from the largest number correctly counted in the forward counting task to one, obtaining one point for each correct response. As well as for forward counting, also in this case we conducted separate analyses from 10 to 1 and from 50 to 11 (expected ranges 0–10 and 0–40, respectively). The test-retest reliability was 0.82 for counting from 10 to 1 and 0.81 for counting from 50 to 11.

##### Digit-quantity mapping

Digit-quantity mapping was assessed using the digit-dots correspondence subtest from the BIN (Molin et al., 2007). In this task, children were asked to match a presented digit ranging from one to nine with the corresponding set of dots among three different visually presented sets, receiving one point for each correct answer (expected range 0–9). The test–retest reliability was.79.

##### Digit naming

Digit naming skills were measured using a task adapted from the number naming subtest of the BIN (Molin et al., 2007), in which participants were shown digits from 1 to 16 and had to read aloud them. One point was given for each digit correctly recognized and named. Analogously to forward and backward counting, we analyzed also digit naming separately for 1–10 and 11–16 (expected ranges 0–10 and 0–6, respectively). The test-retest reliability was 0.86 for naming from 1 to 10 and 0.83 for naming from 11 to 16.

#### Short-Term Memory

To assess STM skills, we used the forward word span task (Lanfranchi et al., 2004). Children were presented with sequences of two to five words and were asked to repeat each list immediately after the presentation in the same order as the examiner. The test included four difficulty levels, for a total of eight trials. A score of one was given for each sequence correctly recalled (expected range 0–8). The test–retest reliability was 0.87.

## Results

Means and standard deviations of scores of the three groups of children are presented in Table 1. Preliminary analysis indicated no difference between the groups in terms of chronological age, *F*(2,147) = 2.67, *p* = 0.07, $\eta_{\text{p}}^{2}$ = 0.035 and gender, *F*(2,147) = 0.006, *p* = 0.99, $\eta_{\text{p}}^{2}$ = 0.000. Therefore, these parameters were not further included as covariates in the analysis.

**TABLE 1:** Mean scores and standard deviations in the different measures of the three groups of children (SRG, YG, and IG).

	Syrian Refugees group (*n* = 47)		Yazidi group (*n* = 48)		Italian group (*n* = 55)	
	*M*	*SD*	*M*	*SD*	*M*	*SD*
STM	5.45	0.97	5.40	1.20	5.00	1.20
WM	1.96	1.76	1.54	1.60	2.49	2.30
Inhibitory control	10.13	2.04	10.10	2.00	13.67	2.44
Forward counting (1–10)	8.89	2.06	8.29	2.90	9.98	0.14
Forward counting (11–50)	6.04	8.95	2.73	6.84	24.24	12.06
Backward counting (10–1)	1.70	3.59	1.83	3.86	5.40	4.93
Backward counting (50–11)	0.28	1.02	0.17	0.72	3.93	10.94
Digit-quantity mapping	4.47	1.92	3.63	2.04	7.16	2.01
Digit naming (1–10)	5.62	2.32	4.50	2.13	9.15	2.11
Digit naming (11–16)	0.21	0.69	0.13	0.53	2.55	2.38

Bivariate correlations between all measured variables are reported in Table 2 for Yazidi and Syrian refugee children and in Table 3 for Italian children. It should be noticed that in all three groups EFs and early math abilities were significantly related.

**TABLE 2:** Bivariate correlations between all variables considered in the study for Yazidi (*n* = 48) and Syrian refugee (*n* = 47) children.

	Zero-order correlations										
	1	2	3	4	5	6	7	8	9	10	11
1. Age (in months)	–	0.42**	0.38**	0.31*	0.44**	0.03	0.21	–0.05	0.38**	0.33*	0.18
2. STM	0.39**	–	0.56***	0.36*	0.16	0.31*	0.38**	0.27	0.29*	0.32*	0.16
3. WM	0.31*	0.01	–	0.76***	0.58***	0.67***	0.71***	0.44**	0.74***	0.72***	0.47***
4. Inhibitory control	0.24	0.12	0.86***	–	0.59***	0.64***	0.76***	0.49***	0.76***	0.78***	0.46***
5. Forward counting (1–10)	0.33*	0.19	0.60***	0.46***	–	0.24	0.29*	0.14	0.60***	0.57***	0.14
6. Forward counting (11–50)	0.20	–0.10	0.90***	0.88***	0.37**	–	0.62***	0.77***	0.66***	0.61***	0.68***
7. Backward counting (10–1)	0.07	–0.14	0.84***	0.75***	0.26	0.89***	–	0.50***	0.70***	0.70***	0.51***
8. Backward counting (50–11)	–0.17	–0.15	0.48***	0.37*	0.15	0.58***	0.64***	–	0.46***	0.50***	0.44**
9. Digit-quantity mapping	0.24	0.10	0.79***	0.77***	0.42**	0.78***	0.77***	0.26	–	0.93***	0.67***
10. Digit naming (1–10)	0.12	0.01	0.73***	0.72***	0.42**	0.75***	0.71***	0.33*	0.91***	–	0.62***
11. Digit naming (11–16)	0.34*	0.21	0.55***	0.51***	0.17	0.50***	0.51***	0.10	0.70***	0.60***	–

* *p* ≤ 0.05, ** *p* ≤ 0.01, *** *p* ≤ 0.001. Yazidis’ results are reported in the upper right part of the table, while Syrian refugees’ results are shown in the lower left part of the table.

**TABLE 3:** Bivariate correlations between all variables considered in the study for Italian children (*n* = 55).

	Zero-order correlations									
	2	3	4	5	6	7	8	9	10	11
1. Age (in months)	–0.02	–0.06	–0.03	–0.12	–0.06	0.06	–0.13	–0.07	0.04	–0.13
2. STM	–	–0.07	–0.12	0.00	0.05	0.03	0.11	0.11	0.05	0.09
3. WM	–	–	0.52***	0.60***	0.58***	0.76***	0.61***	0.60***	0.30*	0.58***
4. Inhibitory control	–	–	–	0.63***	0.44***	0.51***	0.49***	0.57***	0.68***	0.47***
5. Forward counting (1–10)	–	–	–	–	0.42**	0.66***	0.39**	0.91***	0.41**	0.57***
6. Forward counting (11–50)	–	–	–	–	–	0.55***	0.76***	0.51***	0.44***	0.60***
7. Backward counting (10–1)	–	–	–	–	–	–	0.56***	0.68***	0.52***	0.65***
8. Backward counting (50–11)	–	–	–	–	–	–	–	0.44***	0.50***	0.72***
9. Digit-quantity mapping	–	–	–	–	–	–	–	–	0.43***	0.60***
10. Digit naming (1–10)	–	–	–	–	–	–	–	–	–	0.57***
11. Digit naming (11–16)	–	–	–	–	–	–	–	–	–	–

* *p* ≤ 0.05, ** *p* ≤ 0.01, *** *p* ≤ 0.001.

### Group Comparisons

Differences between the three groups of children were investigated through a multivariate analysis of variance (MANOVA) with the group (Syrian Refugees Group-SRG, Yazidi Group-YG, and Italian Group-IG, respectively) used as the fixed factor, and the measures of STM, EFs (i.e., WM and inhibitory control) and early mathematical abilities (i.e., forward and backward counting, digit-quantity mapping, and digit naming) as the dependent variables. Bonferroni-adjusted *post hoc* pair-wise comparisons of scores were also carried out. Univariate test results and Bonferroni’s adjusted *post hoc* pair-wise comparisons from MANOVA between the three groups of children are reported in Table 4.

**TABLE 4:** Univariate test results and Bonferroni’s adjusted *post hoc* pair-wise comparisons from MANOVA between the three groups of children (SRG, YG, and IG).

	*F*	Effect sizes	Groups		*M*_diff_	*p*	*d*
STM	*F*(2,147) = 2.42, *p* = 0.09	0.03	YG	IG	0.40	0.24	0.33
			SRG	IG	0.45	0.15	0.41
			YG	SRG	0.05	1	0.05
WM	*F*(2,147) = 3.14, *p* = 0.046	0.04	YG	IG	–0.095	0.04	0.48
			SRG	IG	–0.053	0.50	0.26
			YG	SRG	–0.042	0.89	0.25
Inhibitory control	*F*(2,147) = 46.16, *p* ≤ 0.001	0.39	YG	IG	–3.57	≤0.001	1.60
			SRG	IG	–3.55	≤0.001	1.57
			YG	SRG	0.02	1	0.01
Forward counting (1–10)	*F*(2,147) = 9.73, *p* ≤ 0.001	0.12	YG	IG	–1.71	≤0.001	0.83
			SRG	IG	–1.11	0.02	0.76
			YG	SRG	0.60	0.43	0.24
Forward counting (11–50)	*F*(2,147) = 74.93, *p* ≤ 0.001	0.51	YG	IG	–21.51	≤0.001	2.19
			SRG	IG	–18.19	≤0.001	1.71
			YG	SRG	–3.31	0.29	0.42
Backward counting (10–1)	*F*(2,147) = 12.97, *p* ≤ 0.001	0.15	YG	IG	–3.57	≤0.001	0.81
			SRG	IG	–3.70	≤0.001	0.86
			YG	SRG	0.13	1	0.03
Backward counting (50–11)	*F*(2,147) = 5.39, *p* = 0.006	0.07	YG	IG	–3.76	0.02	0.49
			SRG	IG	–3.65	0.02	0.47
			YG	SRG	–0.11	1	0.12
Digit-quantity mapping	*F*(2,147) = 44.81, *p* ≤ 0.001	0.38	YG	IG	–3.54	≤0.001	1.74
			SRG	IG	–2.70	≤0.001	1.37
			YG	SRG	0.84	0.12	0.42
Digit naming (1–10)	*F* (2,147) = 64.20, *p* ≤ 0.001	0.47	YG	IG	–4.65	≤0.001	2.19
			SRG	IG	–3.53	≤0.001	1.59
			YG	SRG	–1.12	0.04	0.50
Digit naming (11–16)	*F*(2,147) = 42.49, *p* ≤ 0.001	0.37	YG	IG	–2.42	≤0.001	1.40
			SRG	IG	–2.33	≤0.001	1.34
			YG	SRG	–0.09	1	0.13

*The MANOVA results revealed a significant main effect for group factor [Wilks’ Lambda = 0.31, *F*(20,276) = 11.19, *p* = ≤ 0.001, $\eta_{p}^{2}$ = 0.45], since the three groups (Syrian refugees Group-SRG, Yazidi Group-YG, and Italian Group-IG) significantly differed from each other. To compare differences between groups, $\eta_{p}^{2}$ was used as a measure of effect size. Cohen’s (1988) criteria were used to classify the effect sizes: small effect: $\eta_{p}^{2}$ = 0.01; medium effect: $\eta_{p}^{2}$ = 0.06; and large effect: $\eta_{p}^{2}$ = 0.14. Cohen’s *d* for *post hoc* pairwise comparisons were used as a measure of effect size: small effect *d* = 0.20; medium effect *d* = 0.50; large effect *d* = 0.80.*

Overall, univariate test results established significant differences between the three groups in all measures considered in the study (i.e., EFs and early mathematical abilities), except for STM skills. More specifically, YG and SRG, living in contexts characterized by various conditions of deprivation, showed a lower level of both EFs (i.e., WM and inhibitory control) and early mathematical abilities (i.e., forward and backward counting, digit-quantity mapping, and digit naming) than IG, coming from not deprived sociocultural contexts. Regarding WM, inhibitory control, and early math skills, except for digit naming (1–10), no significant differences were found between the two deprived groups.

## Discussion

The present study grounds on two main aspects enucleated from the previous research: on the one hand, EFs provide a crucial foundation for learning in school settings and early school achievement (see Zelazo et al., 2016), predicting a wide range of important outcomes, such as early math abilities (e.g., Blair and Razza, 2007; Bull et al., 2008; Clark et al., 2010); on the other hand, EFs’ development is critically affected by the levels of stress, disadvantage, or deprivation that children experience early in their lives (Shonkoff et al., 2009). Our results revealed that Yazidi and Syrian children, both coming from highly deprived backgrounds characterized by genocide context and refugee condition respectively, showed poorer EFs skills than Italian preschoolers, thus confirming the link between EFs deficit and exposure to stressful living conditions (e.g., Merz et al., 2016). More in detail, inhibition control resulted more impaired in both Yazidi and Syrian children than Italians, while WM skills emerged as worse only in the sample of Yazidis compared to Italian group. The greater impairment of WM in the Yazidis may be attributed to the fact that the genocide suffered by this latter group represents a condition of extreme and violent deprivation (United Nations Human Rights Council, 2016). There was no significant difference between the three samples of children in STM, used as control measure, in line with recent findings suggesting that STM skills may not be so strongly affected by socio-economic background (Alloway et al., 2017).

Furthermore, the results confirmed the relationship between EFs and early mathematical abilities, already found in typically developing children. Indeed, the two groups of deprived children (i.e., Yazidis and Syrian refugees) not only showed lower EFs skills but also revealed significantly poorer early mathematical abilities than Italians, by performing worse all four mathematical tasks used in the study.

In summary, although all three groups of preschoolers had not yet access a formal approach to the concept of number, it is possible to observe that more deprived children showed significantly lower EFs and poorer early math performance.

Our study is limited in several ways. First of all, we must acknowledge that, although we found correlations between EFs and early math abilities, this does not preclude the possibility that other bio-psycho-social variables could mediate or moderate this relationship. Genetic background (Brett et al., 2015), age differences related to the degree of PFC vulnerability to stressors (McEwen and Morrison, 2013), severity, timing, and duration of deprivation (Beckett et al., 2010) may have contributed to determine our results. Likewise, we have no information regarding certain potentially relevant aspects, such as children’s family composition, difficulties and/or traumas related to prenatal and/or perinatal status, and the possibilities to access to medical care. Furthermore, at a psychological level, differences in domain general cognitive abilities (e.g., intelligence), number words system used, and specific educational stimulations could have driven the data. Lastly, the level of poverty experienced in the specific geopolitical context may have had an impact on the differences we observed in preschool children involved in our study (see Chen et al., 2019).

Secondly, we compared three different situations: Yazidi, Syrian refugee, and Italian preschoolers. The specific environmental and political situation experienced by Yazidi children may not be representative of other different forms of deprivation, such as war and refugee experience, and both the analyzed deprived conditions are substantially dissimilar from the Italian one. In this sense, it would be more methodologically correct to use a sample from the same population but not affected from the crucial political and social events in question (Bos et al., 2009; Lan et al., 2011). In this specific study, anyway, given the extended and complex social situations in the territory, it was not possible to recruit non-affected samples.

It is our belief that this study contributes to the literature in numerous ways: firstly, it is an attempt to evaluate cognitive consequences of genocide and deprivation providing important insight into the effects of these types of experience on both EFs and numerical abilities in early childhood. Secondly, and consistently with the literature, deprivation seems to have an effect on basic abilities, thereby confirming the importance of school-based activities for specific interventions programs. The possibility not only to evaluate but also to apply tailored trainings in these contexts and in other migration-related situations may be crucial for helping future adults deal with the scourge of war.

## Data Availability Statement

The datasets supporting the conclusions of this article are not publicly available due to privacy reason, but are available from the corresponding author on reasonable request.

## Ethics Statement

The studies involving human participants were reviewed and approved by the Region Friuli Venezia Giulia, municipal kindergartens in Trieste, Terre des Hommes. The patients/participants provided their written informed consent to participate in this study.

## Author Contributions

SP designed the scientific work, followed the testing procedure, drafted the first version of the manuscript, and provided a final approval to the manuscript. GA and CD followed the testing procedure, drafted the first version of the manuscript, and provided a final approval to the manuscript. TA critically interpreted the data and provided a final approval to the manuscript. MA designed the scientific work, providing thoughtful advices on the specific socio-political territory where the research took place, and provided a final approval to the manuscript. MP supervised the scientific work, and provided a critical revision of the work and a final approval of the version to be published.

## Conflict of Interest

The authors declare that the research was conducted in the absence of any commercial or financial relationships that could be construed as a potential conflict of interest.
